# Efficacy of Adding Sitagliptin to Ongoing Metformin on Metabolic Profile, Triglyceride-Glucose Index, Vitamin D3, and Liver Tests in Patients Type 2 Diabetes Mellitus and Nonalcoholic Fatty Liver Disease: A Double-Blind Randomized Clinical Trial

**DOI:** 10.1016/j.curtheres.2024.100764

**Published:** 2024-10-16

**Authors:** Habib Yaribeygi, Majid Ramezani, Niki Katsiki, Majid Mirmohammadkhani, Narges Sadat Tabaei

**Affiliations:** 1 Research Center of Physiology, Semnan University of Medical Sciences, Semnan, Iran; 2 Department of Internal Medicine, School of Medicine, Baqiyatallah University of Medical Sciences, Tehran, Iran; 3 Department of Nutritional Sciences and Dietetics, International Hellenic University, Thessaloniki, Greece; 4 School of Medicine, European University Cyprus, Nicosia, Cyprus; 5 Social Determinants of Health Research Center, Semnan University of Medical Sciences, Semnan, Iran

**Keywords:** diabetes mellitus, glucagon-like peptide 1, liver function test, nonalcoholic fatty liver disease, sitagliptin

## Abstract

**Background:**

Dipeptidyl peptidase-4 inhibitors provide potent antidiabetic effects in patients with type 2 diabetes mellitus (T2DM), but their role in the presence of nonalcoholic fatty liver disease (NAFLD) is not well-known.

**Objective:**

The aim of this clinical trial was to evaluate the effects of sitagliptin on the metabolic profile and liver test results in metformin-treated patients with T2DM and NAFLD.

**Methods:**

This was a prospective, 12-week, single-center, comparative randomized clinical trial enrolling 66 adult patients with T2DM and NAFLD (diagnosed by ultrasound). Patients were randomly assigned to either metformin (2000 mg/d, n = 33) or sitagliptin + metformin (100 and 2000 mg/d, respectively, n = 33), administered orally. Certain metabolic parameters, that is, fasting blood sugar (FBS), glycosylated hemoglobin, triglycerides (TGs), total cholesterol (TC), low-density lipoprotein cholesterol, vitamin D3 (vitD3), alkaline phosphatase, alanine aminotransferase (SGPT), and aspartate aminotransferase, were measured at baseline and after 12 weeks. Triglyceride-glucose (TG-G) index was also calculated.

**Results:**

All biochemical variables decreased by a greater extent in the sitagliptin + metformin group than in the metformin group, with differences in FBS (*P* = 0.030), TC (*P* = 0.017), TG (*P* = 0.008), SGPT (*P* = 0.018), and vitD3 (*P* = 0.001) reaching statistical significance. Furthermore, the mean reduction of the TG-G index was significantly greater in the sitagliptin + metformin group than in the metformin group (0.67 vs 0.21, respectively; *P* = 0.017).

**Conclusions:**

Sitagliptin + metformin therapy led to significantly greater improvements in FBS, TC, TG, SGPT, vitD3, and TG-G compared with the metformin monotherapy group. Other biomarkers also decreased more in the sitagliptin + metformin group than in the metformin group, but these differences did not reach statistical significance. The present findings should be interpreted with caution, although they suggest certain metabolic benefits after sitagliptin addition in metformin-treated patients with T2DM and NAFLD. Further studies are required to elucidate these effects and provide strong evidence for safe conclusions.

## Introduction

Diabetes mellitus (DM) is the most common metabolic disorder globally.^1^^,^^2^ In 2021, the number of patients with DM was 366 million worldwide, which is estimated to increase to approximately 522 million by 2030.^2^ Consequently, the annual costs of DM prevention and treatment are gradually increasing.^3^ Diabetes mellitus is classically divided into 3 types as: type 1, type 2 DM (T2DM), and gestational DM.^1^^,^^2^ Type 2 DM is the more prevalent one (up to 95% of all cases), and it is mainly characterized by insulin resistance (ie, lack of response to circulating insulin) in peripheral tissues, hyperglycemia, and hyperinsulinemia.^1^ The increased levels of blood glucose negatively affect the function of several organs, especially the eyes, kidneys, nerves, and cardiovascular (CV) system, leading to the development of microvascular and macrovascular diabetic complications.^4^

Type 2 DM is a potent risk factor for hepatic disorder, involving a series of liver complications such as cirrhosis and fibrosis, hepatocellular carcinoma, acute liver failure, hepatitis, bile duct diseases, and nonalcoholic fatty liver.^5^^,^^6^ Nonalcoholic fatty liver disease (NAFLD) is the most frequent form of chronic liver disease, caused by excess fat accumulation within the hepatocytes, usually developed in obese or overweight people in the absence of alcohol abuse.^7^ This hepatic disorder involves a spectrum of liver abnormalities from nonalcoholic fatty liver to nonalcoholic steatohepatitis (NASH), progressively also leading to cirrhosis and hepatocellular cancer.^7^ Therefore, the early diagnosis and adequate management of NAFLD, as well as its prevention, are of paramount importance in order to protect against further liver complications.^7^ Strong evidence demonstrates that T2DM is closely associated with the pathophysiology of NAFLD, and thus, NAFLD and NASH have an increased prevalence in patients with T2DM compared with nondiabetic populations.^7^^,^^8^ NAFLD has also been related to increased CV morbidity and mortality.^9^ There is a growing body of evidence supporting a link between NAFLD/NASH and vitamin D3 (vitD3) deficiency.^10^ This vitamin is also involved in glucose and lipid metabolism,^11^ as well as in liver function.^12^

Glucagon-like peptide-1 (GLP-1) is an incretin hormone produced by the L-cells of the enterocytes and released into the circulation to provide hypoglycemic effects via several pathways.^13^ This peptide is physiologically inactivated by the dipeptidyl peptidase-4 (DPP-4) enzyme; DPP-4 inhibitors have been developed as antidiabetic drugs that are able to increase GLP-1 half-life to prolong its physiologic activities and glucose-lowering effects.^13^ Sitagliptin is a DPP-4 inhibitors indicated for T2DM management, but there is limited data on its effects on NAFLD (mainly neutral).^14^^,^^15^ Recently, a clinical trial reported that 24 weeks of sitagliptin may be able to improve liver efficiency without benefits on liver lipids in patients with NAFLD.^16^ Another recent trial also suggested that sitagliptin (in 56 weeks) improves liver function tests in patients with NAFLD.^17^

The aim of the present clinical trial was to examine the effects of 12 weeks of sitagliptin on metabolic parameters, including the triglyceride-glucose (TG-G) index, vitD3, and liver test results in patients with T2DM and NAFLD.

## Patients and Methods

This was a 12-week, double-blind, randomized, controlled trial involving patients with T2DM and NAFLD (Clinical Trial Registration: https://www.irct.ir/trial/61583). In detail, 66 patients with T2DM (40–65 years of age) and NAFLD (based on criteria defined in the study by Burt et al^18^) referred to the endocrinology clinic (Mir Emad Street, Tehran, Iran) were screened for participation in this study from February to September 2022. Inclusion criteria were (1) having T2DM (defined as glycosylated hemoglobin [HbA_1c_] ≥6.8%, fasting blood sugar [FBS] ≥126 mg/dL, and 2-hour postprandial glucose ≥200 mg/dL); (2) having NAFLD (diagnosed by liver ultrasound); and (3) have given informed consent to participate in the study.

Exclusion criteria were: taking GLP-1 analogues, history of malignancy, AIDS, hepatitis, chronic kidney disease, CV disease, hemoglobinopathies or other conditions that may affect HbA_1c_, active pancreatitis, active viral or bacterial infection, active inflammatory or autoimmune disorders, taking drugs that may influence glucose metabolism (eg, glucocorticoids or immunosuppressive), pregnancy/breastfeeding, or taking viral agents. Patients were also excluded if any allergic reactions or side effects occurred during the study follow-up, or if any acute disease was developed that led to the discontinuation of the treatment or needing further drugs. The patients were randomly assigned into either sitagliptin + metformin (100 mg + 2000 mg/d, n = 29) or metformin alone (2000 mg/d, n = 31), according to a computer-generated randomization sequence.

Randomization was performed using the stratified permuted block randomization method. The classification variable was gender and square blocks were used. Patients were advised to take 2 tablets daily (metformin: 2 × 1000 mg, sitagliptin + metformin: 2 × 50 mg/1000 mg, respectively), one after breakfast and one after dinner, without any changes in dietary or other drug intake or physical activity during the trial. The patient and the analyzer of the final results were blind to the grouping, but the attending physician and the principal investigator were aware.

After a minimum of 8 hours of overnight fasting, a 10 mL blood sample was collected for assessment of biochemical factors as FBS, HbA_1c_, TG, total cholesterol (TC), low-density lipoprotein (LDL) cholesterol, high-density lipoprotein cholesterol, alkaline phosphatase (ALP), alanine aminotransferase (SGPT), aspartate aminotransferase (SGOT), and vitD3. Triglyceride-glucose index was also calculated as ln[TG*FBS/2].^19^ The patients were followed up for 12 weeks; all measurements were performed at baseline and at 12 weeks. The primary outcome measure in this trial was the reduction in FBS between metformin and metformin + sitagliptin groups. Also, the secondary endpoints of this trial included improvement in glycemic, lipidemic, and liver function profiles.

### Sample size calculation

In this study, the primary outcome was the reduction of alanine transaminase (ALT) in patients. To calculate the sample size, the results of the study by Ohki et al^20^ were used. Based on the calculated effect size, which confirms the high effect of the drug on ALT (d > 2), using GPower software (GPower 3.1) for each group and 80% power and 95% confidence for equal groups, 2-domain *t* test was performed, and to compare the means considering the effective size effect (d = 0.9), the sample size was calculated to be 66 participants.

### Statistical analysis

Mean and SD were used to report quantitative variables, except that median (range) was used to present non-normal values. The normality of data distribution was checked using the Shapiro–Wilk test (Table 1). Independent *t* test was used in the comparison between 2 groups in case of normally distributed continuous data. Paired *t* test is used in comparison within each group in case of normally distributed continuous data. Mann–Whitney *U* test was used in the comparison between 2 groups in case of nonnormally distributed continuous data. Wilcoxon signed-rank test was used in comparison within each group in case of nonnormally distributed continuous data. Spearman's correlation test was used to check the correlation between some quantitative variables of interest. Based on this, the baseline values and comparisons of laboratory parameters of FBS, TC, TG, LDL cholesterol, SGOT, SGPT, HbA_1c_, vitD3, and ALP at baseline and at 12 weeks in each group were analyzed via the Mann–Whitney *U* test method. However, comparisons of changes in the biochemical variables within and between groups were performed via the Wilcoxon signed-rank test. Also, comparisons of TG-G index and its changes during the study within and between the study groups were performed by paired *t* test. All statistical analysis was performed using the SPSS software version 25.0 (SPSS Inc, Chicago, Illinois). A 2-sided *P* value <0.05 was considered significant for all statistical tests.

**Table 1:** Results of normality test using Shapiro–Wilk test.

	Group	Kolmogorov–Smirnov*			Shapiro–Wilk		
		Statistics	*df*	Sig.	Statistics	*df*	Sig.
Age	Metformin	0.127	31	0.200†	0.970	31	0.526
	Sitagliptin + metformin	0.099	26	0.200†	0.958	26	0.347

Sig. = significance.

⁎ Lilliefors significance correction.

† This is a lower bound of the true significance.

### Ethics

This clinical trial was conducted in accordance with the Helsinki Declaration and the local ethical instructions, and it has been approved by the Research Ethics Committees of Semnan University of Medical Sciences and Health Services (IR.SEMUMS.REC.1400.303) and the Iranian Registry of Clinical Trials (IRCT20191209045671N2, available at: https://www.irct.ir/trial/61583). The protocol was clearly explained orally to all participants, and their informed written consent was obtained before their inclusion into the study. All patients were monitored closely during the study being in contact with the researchers and physicians throughout the study.

## Results

Sixty patients (31 in the metformin group and 29 in the sitagliptin + metformin group) completed the trial. Six randomly assigned patients who did not complete the study either discontinued by their own decision or were discontinued by the study physician, leaving a total of 60 patients who completed the trial (Figure). Also, 2 patients reported vomiting as a side effect of sitagliptin. So, analyses were based on observed data only, and no excess data was imputed.

**Fig:**
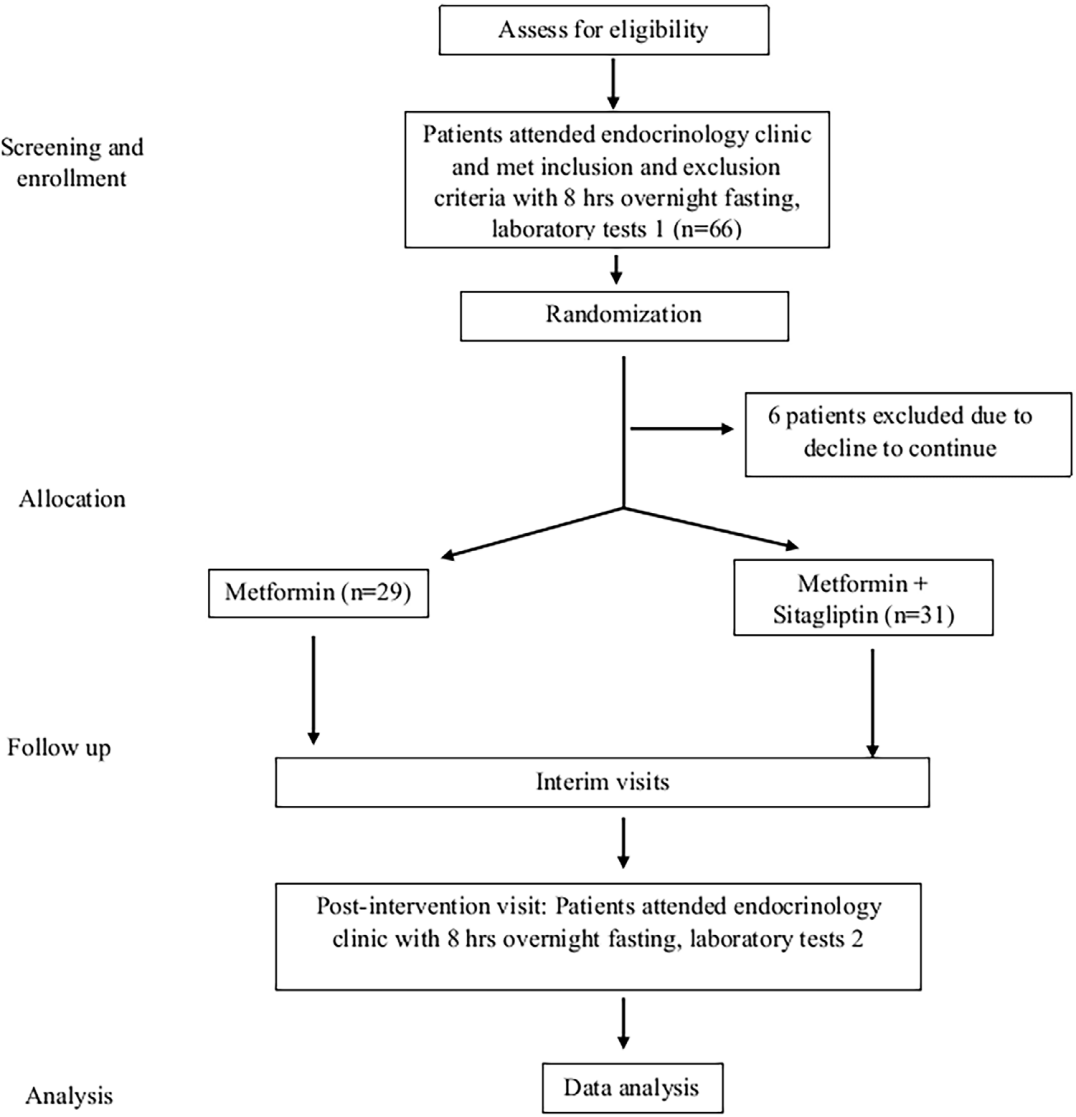
Study flowchart based on consolidated standards of reporting trials (CONSORT) diagram.

Age and gender did not significantly differ between the 2 study groups, although they were not the same, and so were analyzed via χ^2^ and *t* test methods (Table 2). However, other evaluated factors did not have normal distribution and thus were analyzed via a nonparametric test of Mann–Whitney *U* test. Metabolic data at baseline are presented in Table 3. Comparisons of laboratory variables at baseline and 12 weeks in each study group are shown in Table 4. At baseline, FBS (*P* = 0.019) and TG (*P* = 0.044) were significantly higher in the sitagliptin + metformin group than in the metformin group (for FBS, 154 ± 47 vs 133 ± 35 mg/dL; *P* = 0.019; for TG, 254 ± 135 vs 177 ± 80 mg/dL; *P* = 0.044, respectively). At 12 weeks of therapy, TC and ALP were significantly lower in the sitagliptin + metformin group than in the metformin group (for TC, 136 ± 31 vs 163 ± 43 mg/dL; *P* = 0.026; for ALP, 152 ± 26 vs 207 ± 42 IU/L; *P* = 0.025, respectively).

**Table 2:** Gender and age of participants in each study group.

*P*	Sitagliptin + metformin group (n = 29)	Metformin group (n = 31)	Variables
0.599*	13/16	16/15	Gender (M/F)
0.295†	54.3 (9.2)	51.9 (7.3)	Age (y), mean (SD)

F = female; M = male.

⁎ χ^2^ test.

† *t* test.

**Table 3:** The baseline metabolic data of each group.

Index	Group	Mean (SD)	P*
FBS	Metformin	132.630 (35.3946)	0.019
	Sitagliptin + metformin	153.640 (46.6663)	
CHOL	Metformin	178.800 (48.7837)	0.337
	Sitagliptin + metformin	194.350 (68.4715)	
TG	Metformin	177.318 (79.9901)	0.044
	Sitagliptin + metformin	253.591 (134.8543)	
LDL cholesterol	Metformin	91.000 (37.3631)	0.479
	Sitagliptin + metformin	108.333 (61.2096)	
SGOT	Metformin	30.480 (10.7553)	0.208
	Sitagliptin + metformin	35.036 (15.6784)	
SGPT	Metformin	39.320 (12.4757)	0.123
	Sitagliptin + metformin	45.643 (16.5153)	
HbA_1c_	Metformin	6.577 (0.9091)	0.706
	Sitagliptin + metformin	6.858 (1.4134)	
VitD3	Metformin	30.920 (7.4898)	0.291
	Sitagliptin + metformin	27.606 (10.5859)	
ALK	Metformin	224.400 (22.2216)	0.841
	Sitagliptin + metformin	241.556 (94.2206)	

FBS = fasting blood sugar; HbA_1c_ = glycosylated hemoglobin; LDL = low-density lipoprotein; SGOT = aspartate aminotransferase; SGPT = alanine aminotransferase; TG = triglyceride; vitD3 = vitamin D3; ALK = alkaline phosphatase; Chol = cholesterol.

⁎ Mann–Whitney *U* test.

**Table 4:** Comparisons of laboratory parameters at baseline and 12 weeks in each group.

Variables	Time	Group	Mean (SD)	P*
FBS (mg/dL)	Baseline	Metformin	133 (35)	0.019
		Sitagliptin + metformin	154 (47)	
	12 wk	Metformin	120 (17)	0.052
		Sitagliptin + metformin	114 (32)	
TC (mg/dL)	Baseline	Metformin	179 (49)	0.337
		Sitagliptin + metformin	194 (68)	
	12 wk	Metformin	163 (43)	0.026
		Sitagliptin + metformin	136 (31)	
TG (mg/dL)	Baseline	Metformin	177 (80)	0.044
		Sitagliptin + metformin	254 (135)	
	12 wk	Metformin	148 (62)	0.874
		Sitagliptin + metformin	161 (77)	
LDL cholesterol (mg/dL)	Baseline	Metformin	91 (37)	0.479
		Sitagliptin + metformin	108 (61)	
	12 wk	Metformin	84 (34)	0.139
		Sitagliptin + metformin	70 (26)	
SGOT (IU/L)	Baseline	Metformin	30 (11)	0.208
		Sitagliptin + metformin	35 (16)	
	12 wk	Metformin	25 (8)	0.468
		Sitagliptin + metformin	26 (9)	
SGPT(IU/L)	Baseline	Metformin	39 (12)	0.123
		Sitagliptin + metformin	46 (16)	
	12 wk	Metformin	36 (13)	0.333
		Sitagliptin + metformin	33 (12)	
HbA_1c_ (%)	Baseline	Metformin	6.6 (0.9)	0.706
		Sitagliptin + metformin	6.8 (1.4)	
	12 wk	Metformin	6.3 (0.7)	0.207
		Sitagliptin + metformin	6.2 (0.8)	
VitD3 (IU/L)	Baseline	Metformin	30.9 (7.5)	0.291
		Sitagliptin + metformin	27.6 (10.6)	
	12 wk	Metformin	33.2 (7.2)	0.200
		Sitagliptin + metformin	41.9 (15.8)	
ALP (IU/L)	Baseline	Metformin	224 (22)	0.841
		Sitagliptin + metformin	241 (94)	
	12 wk	Metformin	207 (42)	0.025
		Sitagliptin + metformin	152 (26)	

ALP = alkaline phosphatase; FBS = fasting blood glucose; HbA_1c_ = glycosylated hemoglobin; LDL = low-density lipoprotein; SGOT = Serum Glutamic Oxaloacetic Transaminase (Aspartate Aminotransferase (AST)); SGPT = Serum Glutamic Pyruvic Transaminase (Alanine Aminotransferase (ALT)); TC = total cholesterol; TG = triglyceride; vitD3 = vitamin D3.

⁎ Mann–Whitney *U* test.

Table 5 shows the comparisons of changes in biochemical variables during the 12-week period within and between the 2 study groups. All variables were improved in each study group, except for LDL cholesterol (in both groups), ALP (in both groups), HbA_1c_ and vitD3 (only in the metformin group), thus indicating that the addition of sitagliptin was responsible for further reductions in HbA_1c_ and vitD3, leading to significant changes only in the sitagliptin + metformin. Furthermore, all biochemical variables decreased by a greater extent in the sitagliptin + metformin group than in the metformin group, and these differences in the effectiveness of therapy were statistically significant for FBS, TC, TG, SGPT, and vitD3 (Table 5).

**Table 5:** Comparisons of changes in the biochemical variables within and between groups.

Change (Week 12 minus Baseline)		Median	Range		P*	*P*†
			Min	Max		
FBS (mg/dL)	Metformin	13	3	24	0.011	0.030
	Sitagliptin + metformin	38	13	63	0.002	
TC (mg/dL)	Metformin	17	0.5	35	0.009	0.017
	Sitagliptin + metformin	62	24	99	<0.001	
TG (mg/dL)	Metformin	34	10	58	0.003	0.008
	Sitagliptin + metformin	101	35	168	<0.001	
LDL cholesterol (mg/dL)	Metformin	6	6	19	0.135	0.095
	Sitagliptin + metformin	32	2	62	0.073	
SGOT (IU/L)	Metformin	7	4	10	<0.001	0.925
	Sitagliptin + metformin	9	2	15	0.002	
SGPT (IU/L)	Metformin	4	0.4	8	0.041	0.018
	Sitagliptin + metformin	14	7	21	<0.001	
HbA_1c_ (%)	Metformin	0.2	0.02	0.5	0.121	0.131
	Sitagliptin + metformin	0.7	0.1	1.3	0.013	
VitD3 (IU/L)	Metformin	2.3	6.3	1.7	0.221	0.001
	Sitagliptin + metformin	4.3	21	7.8	0.002	
ALP (IU/L)	Metformin	12	13	37	0.180	0.083
	Sitagliptin + metformin	120	227	467	0.109	

ALP = alkaline phosphatase; FBS = fasting blood glucose; HbA_1c_ = glycosylated hemoglobin; LDL = low-density lipoprotein; SGOT = Serum Glutamic Oxaloacetic Transaminase (Aspartate Aminotransferase (AST); SGPT = Serum Glutamic Pyruvic Transaminase (Alanine Aminotransferase (ALT); TC = total cholesterol; TG = triglyceride; vitD3 = vitamin D3.

⁎ Wilcoxon signed ranks test (within a group).

† Mann–Whitney *U* test (between groups).

In terms of TG-G index, patients in the sitagliptin + metformin group had an increased value at baseline compared with the metformin group (*P* = 0.003) (Table 6). This index was significantly lower in both study groups (*P* = 0.003 for the metformin group and *P* = 0.001 for the sitagliptin + metformin group). Mean change in TG-G was significantly greater in the sitagliptin + metformin group than in the metformin group (*P* = 0.017), indicating that sitagliptin + metformin was more effective in reducing this index (Table 6).

**Table 6:** Comparisons of triglyceride-glucose index and its changes during the study within and between the study groups.

Group	Baseline	12 wk	Change	P*
	Mean (SD)	Mean (SD)	Mean (SD)	
Metformin	9.18 (0.49)	9.02 (0.48)	0.21 (0.29)	0.003
Sitagliptin + metformin	9.71 (0.60)	8.99 (0.56)	0.67 (0.66)	0.001
*P*†	0.003	0.896	0.017	-

⁎ Paired *t* test.

† *t* test.

## Discussion

In the present study, among 60 patients with T2DM and NAFLD, changes from baseline at week 12 in FBS, TC, TG, SGPT, and vitD3 were significantly greater in the sitagliptin + metformin compared with the metformin group. All other biomarkers also decreased more in the sitagliptin + metformin group than in the metformin group, but these differences did not reach statistical significance. Interestingly, TG-G index was significantly lowered in both groups, but significantly more in the combination group.

The finding that sitagliptin can further lower FBS compared with metformin monotherapy is in accordance with previous studies reporting that sitagliptin strengthens the metformin-induced glucose-lowering effects in patients with T2DM.^21^ There is no evidence reporting the exact mechanism, but it may be due to combination therapy and adding the effects of these therapies in these patients. Recent evidence suggests that increased FBS levels can act as a potent inducer of NAFLD,^22^ even in non-obese people with normal lipid profile,^23^ thus representing an independent NAFLD risk factor.^24^ Chronic fasting hyperglycemia induces adipocyte deposition in hepatic cells toward steatosis development and predicts impaired liver function in the long term.^22^^,^^25^ Furthermore, it can induce and promote pathophysiologic pathways, such as oxidative stress and aberrant inflammation, which are highly involved in liver injuries.^26^ Moreover, insulin resistance is another independent risk factor for NAFLD, leading to its development through several pathways.^27^ There is no strong evidence shedding light on involved mechanisms, but we suggest that FBS is a main marker of metabolic profile, pancreatic function during fasting, and peripheral insulin sensitivity. Therefore, it could translate into long-term glucose and lipid levels, which in turn affect liver sufficiency. Overall, normalizing FBS levels (which is dependent on insulin sensitivity and glucose homeostasis) is highly important in order to prevent T2DM-induced liver dysfunction.^22^^,^^28^ We reported in the present study that sitagliptin + metformin was more effective in reducing FBS at 12 weeks compared with metformin monotherapy. Sitagliptin + metformin also significantly decreased HbaA_1c_. Similar findings were reported in a recent study involving 68 patients with T2DM and NAFLD; sitagliptin therapy for 24 weeks significantly decreased FBS and HbA_1c_.^17^

In the present study, in addition to glucose homeostasis, sitagliptin also further improved dyslipidemia (ie, reduced TC and TG), another risk factor for NAFLD development. Indeed, dyslipidemia can increase the deposition of lipid droplets in the hepatocytes and intensify steatosis toward severe degrees of tissue damage such as fibrosis and necrosis.^9^ Along with hyperglycemia and glucotoxicity, dyslipidemia can result in liver fat deposition by stimulating the oxidation of surrounding adipose tissue, thus leading to increased levels of circulating free fatty acids which in turn intensifies insulin resistance and free radical production.^17^ All the above processes lead to a vicious cycle of worsening lipotoxicity, liver fat accumulation, and insulin resistance.^29^ Therefore, normalizing the lipid profile is another strategy to prevent liver disorders,^30^ as well as to minimize CV risk in patients with NAFLD with or without T2DM.^31^^,^^32^ Previous studies have also reported that sitagliptin improves the lipid profile of patients with T2DM.^29^^,^^33^ Nevertheless, there is a need for further evidence in this field.

Vitamin D3 is recognized as an effective modulator of several metabolic pathways, as well as insulin sensitivity.^11^^,^^34^ Furthermore, its circulating levels have been negatively related to the risk of NAFLD development.^10^^,^^35^ In the present study, the observed changes in vitD3 levels were significantly greater in the sitagliptin + metformin group compared with metformin monotherapy, thus highlighting the ability of sitagliptin to further increase circulating vitD3 concentrations. To the best of our knowledge, this is the first study reporting evidence of raising vitD3 by sitagliptin in patients with T2DM and NAFLD, and the mediating molecular pathways are not clear. It may be due to improvement in liver function by sitagliptin or lowering the oxidative stress which destroys vitD3.^36^ Previous studies have reported that most patients with T2DM have lower vitD3 levels compared with the general population,^37^ and this can negatively affect the metabolic pathways of lipids and glucose, as well as liver function.^11^^,^^34^^,^^38^ Therefore, the potentiality of sitagliptin to increase vitD3 levels can become clinically important in patients with T2DM.

In the present study, both study groups significantly reduced TG-G index, but the sitagliptin + metformin group achieved significantly greater improvements. To the best of our knowledge, this is the first study examining the possible benefits of sitagliptin therapy on TG-G index in patients with T2DM and NAFLD. Further, larger studies with longer duration are required to elucidate these effects.

In terms of liver tests, sitagliptin + metformin therapy for 12 weeks led to significant decreases in SGOT and SGPT (but not ALP); SGPT reduction was significantly more marked in the sitagliptin + metformin compared with the metformin monotherapy group. There is limited evidence from previous studies that sitagliptin can reduce the levels of parameters of liver tests (including SGPT, SGOT, Gamma-glutamyl Transferase (gammaGT)) in patients with NAFLD and even improve histologic features of NAFLD/NASH),^16^^,^^17^^,^^39^ but further research is needed in the field. However, it may be due to improved metabolic status and lipid profile; which in turn improves liver function.^40^ Of note, no certain drug has been approved for NAFLD/NASH treatment with lifestyle intervention currently representing the first-line therapy in such patients.^41^ Nutraceuticals may also prove beneficial.^42^ Nevertheless, hypolipidemic, antiobesity and GLP-1 analogues have been reported to improve liver test results and histology in patients with NAFLD/NASH,^43^^,^^44^ but there is still no official indication for treating this disease with such drugs.

The present study had certain strengths, including the study design (ie, randomized, double-blind, controlled clinical trial) and the evaluation of vitD3 and TG-G index. However, it had some limitations, including the small sample size, the relatively short duration (ie, 12 weeks), and the lack of liver biopsies (which are the “gold standard” for NAFLD/NASH diagnosis). Also, no attempt was made to control for false positive results (type 1 error). Moreover, we acknowledge that we did not consider significant confounding factors when interpreting the results.

## Conclusion

Among 60 patients with T2DM and NAFLD, sitagliptin + metformin therapy led to significantly greater improvements in FBS, TC, TG, SGPT, vitD3, and TG-G index compared with the metformin monotherapy group. Other biomarkers also decreased more in the sitagliptin + metformin group than in the metformin group, but these differences did not reach statistical significance. The present findings cannot be extrapolated to all populations, and thus, they should be interpreted with caution. Further studies are required to elucidate the effects of adding sitagliptin in metformin-treated patients with T2DM and NAFLD.

## Funding

This trial was supported by a fund provided by the Deputy of Research and Technology of Semnan University of Medical Sciences (number 1967). Furthermore, it was supported by a grant provided by Abidi Pharmaceuticals (number 109381).

## Declaration of competing interest

The authors declare that we don't have any conflict of interest in this study.
